# Clinical trials for treating recurrent head and neck cancer with boron neutron capture therapy using the Tsing-Hua Open Pool Reactor

**DOI:** 10.1186/s40880-018-0295-y

**Published:** 2018-06-19

**Authors:** Ling-Wei Wang, Yen-Wan Hsueh Liu, Fong-In Chou, Shiang-Huei Jiang

**Affiliations:** 1 Department of Oncology, Taipei Veterans General Hospital, No. 201, Section 2, Shih-Pai Road, Taipei, 11217 Taiwan China; 2 School of Medicine, National Yang Ming University, Taipei, 11217 Taiwan China; 3 Institute of Nuclear Engineering and Science, National Tsing Hua University, Hsin-Chu, Taiwan China; 4 Nuclear Science and Technology Development Center, National Tsing Hua University, Hsin-Chu, Taiwan China

**Keywords:** Head and neck cancer, Boron neutron capture therapy, Tsing-Hua Open Pool Reactor, Boronophenylalanine

## Abstract

Head and neck (HN) cancer is an endemic disease in Taiwan, China. Locally recurrent HN cancer after full-dose irradiation poses a therapeutic challenge, and boron neutron capture therapy (BNCT) may be a solution that could provide durable local control with tolerable toxicity. The Tsing-Hua Open Pool Reactor (THOR) at National Tsing-Hua University in Hsin-Chu, provides a high-quality epithermal neutron source for basic and clinical BNCT research. Our first clinical trial, entitled “A phase I/II trial of boron neutron capture therapy for recurrent head and neck cancer at THOR”, was carried out between 2010 and 2013. A total of 17 patients with 23 recurrent HN tumors who had received high-dose photon irradiation were enrolled in the study. The fructose complex of l-boronophenylalanine was used as a boron carrier, and a two-fraction BNCT treatment regimen at 28-day intervals was used for each patient. Toxicity was acceptable, and although the response rate was high (12/17), re-recurrence within or near the radiation site was common. To obtain better local control, another clinical trial entitled “A phase I/II trial of boron neutron capture therapy combined with image-guided intensity-modulated radiotherapy (IG-IMRT) for locally recurrent HN cancer” was initiated in 2014. The first administration of BNCT was performed according to our previous protocol, and IG-IMRT was initiated 28 days after BNCT. As of May 2017, seven patients have been treated with this combination. The treatment-related toxicity was similar to that previously observed with two BNCT applications. Three patients had a complete response, but locoregional recurrence was the major cause of failure despite initially good responses. Future clinical trials combining BNCT with other local or systemic treatments will be carried out for recurrent HN cancer patients at THOR.

## Background

Head and neck (HN) cancer has a high frequency worldwide [1], but in Taiwan, China, squamous cell carcinoma (SCC) of the oral cavity is an endemic disease due to the habit of chewing betel nuts. In 2014, more than 7000 individuals were diagnosed with HN cancer [2]. Despite progress in multimodality treatment for this disease, locoregional recurrence is common and poses a therapeutic challenge, especially for patients who already received high-dose photon irradiation [3–5]. Consequently, there is a pressing need for other treatment options for recurrent HN cancer patients.

Boron neutron capture therapy (BNCT) is a type of particle radiotherapy based on the nuclear capture and fission reactions that occur when non-radioactive boron (^10^B) is irradiated with thermal neutrons (< 1 eV) to yield high-energy alpha-particles (^4^He) and recoiling lithium (^7^Li) nuclei. The path-lengths of these particles are approximately one cell diameter making BNCT a potentially ideal way to selectively destroy malignant cells and spare normal tissues if a sufficiently high concentration of ^10^B is localized in tumor cells [6]. Based on a literature review, high response rates (60–83%) and low toxicity rates were reported after BNCT for recurrent HN cancer in both single- and two-fraction treatment protocols [7–9]. As reported a more recent Finnish study, only one had grade 4 toxicity among 29 evaluated patients who received BNCT. The incidence of grade 3 toxicity ranged from 20% to 54%. BNCT for recurrent HN cancer patients was initiated in Taiwan, China in 2010 based on the Japanese and Finnish studies [7–9].

**Fig. 1:**
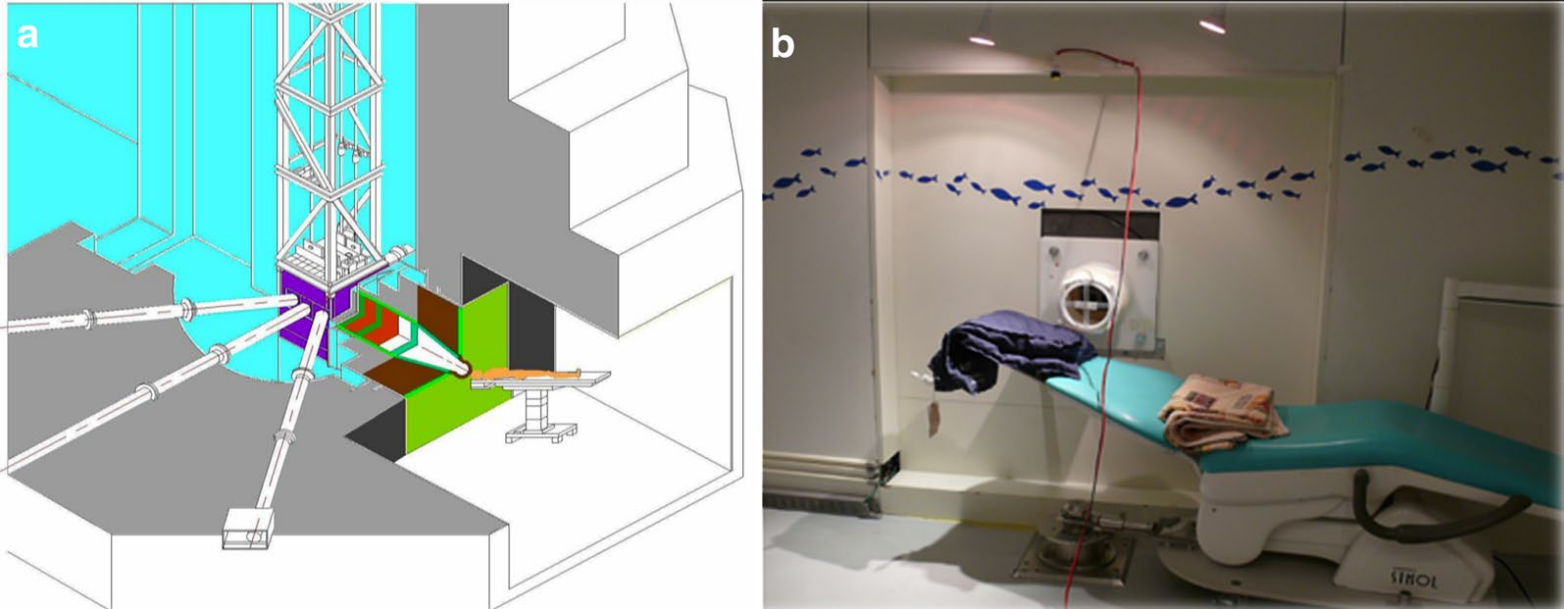
A view of the Tsing-Hua Open Pool Reactor (THOR) and a photo of the treatment room. **a** An image of THOR; the treatment room for boron neutron capture therapy (BNCT) is at the lower right corner. **b** The treatment couch and polyethylene extension collimator in the THOR irradiation room for maintaining treatment position during epithermal neutron irradiation

**Fig. 2:**
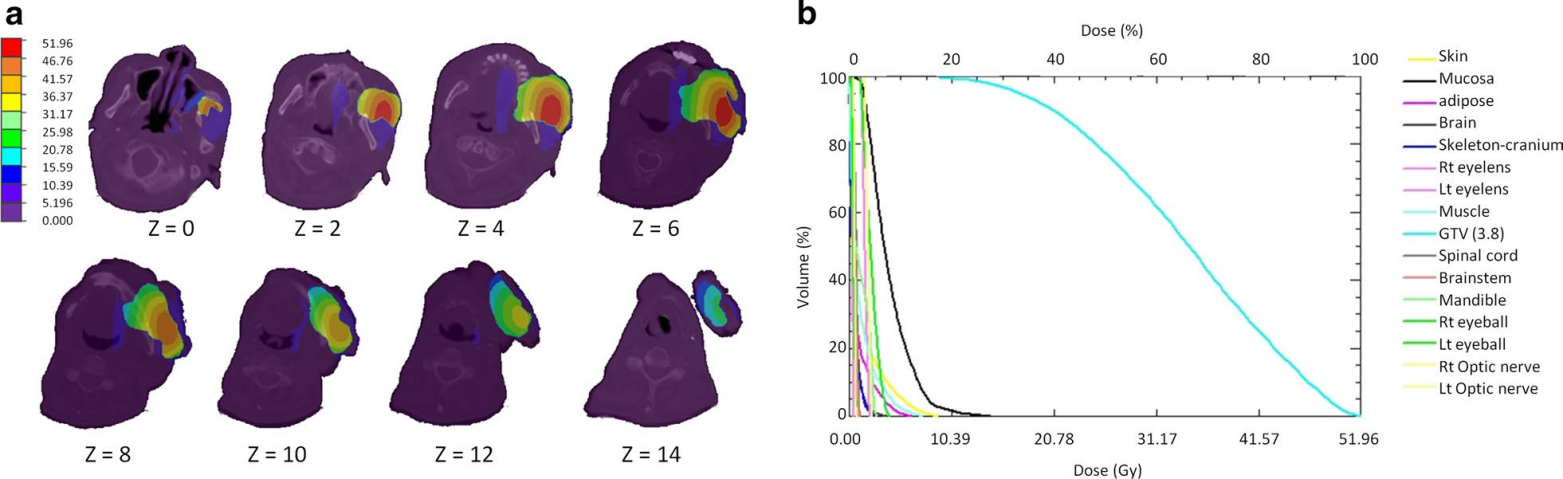
Dose distribution and dose volume histogram (DVH) calculated by THORplan for one patient treated with fractionated boron neutron capture therapy. **a** Three-dimensional dose distribution for a man with recurrent buccal cancer by THORplan. **b** DVH for the same case by THORplan. The gross tumor volume (light blue, right) received far more of the dose than normal tissues (other colors)

The National Tsing-Hua University has a 2-MW open pool research reactor [THOR]; manufactured by General Atomics, San Diego, CA, USA, which is the only epithermal neutron source for BNCT research in Taiwan, China. As a first step, we designed neutron beam and developed a treatment planning system (TPS) for clinical applications [10–12]. In 2004, the construction of our BNCT treatment facility was completed. The beam provided epithermal neutron fluxes > 1.0 × 10^9^ n/cm^2^/s with very low fast neutron and gamma ray contamination (Fig. 1). Later, the beam quality was measured and these data were imported into the TPS (THORplan), which provided 3-dimensional dose displays on computed tomographic (CT) images, and generated dose volume histograms for both the tumor and normal structures [11, 13] (Fig. 2). The purpose of this review is to summarize our clinical experience in treating patients with locally recurrent HN cancer at THOR.

## First clinical trial (2010–2013)

**Fig. 3:**
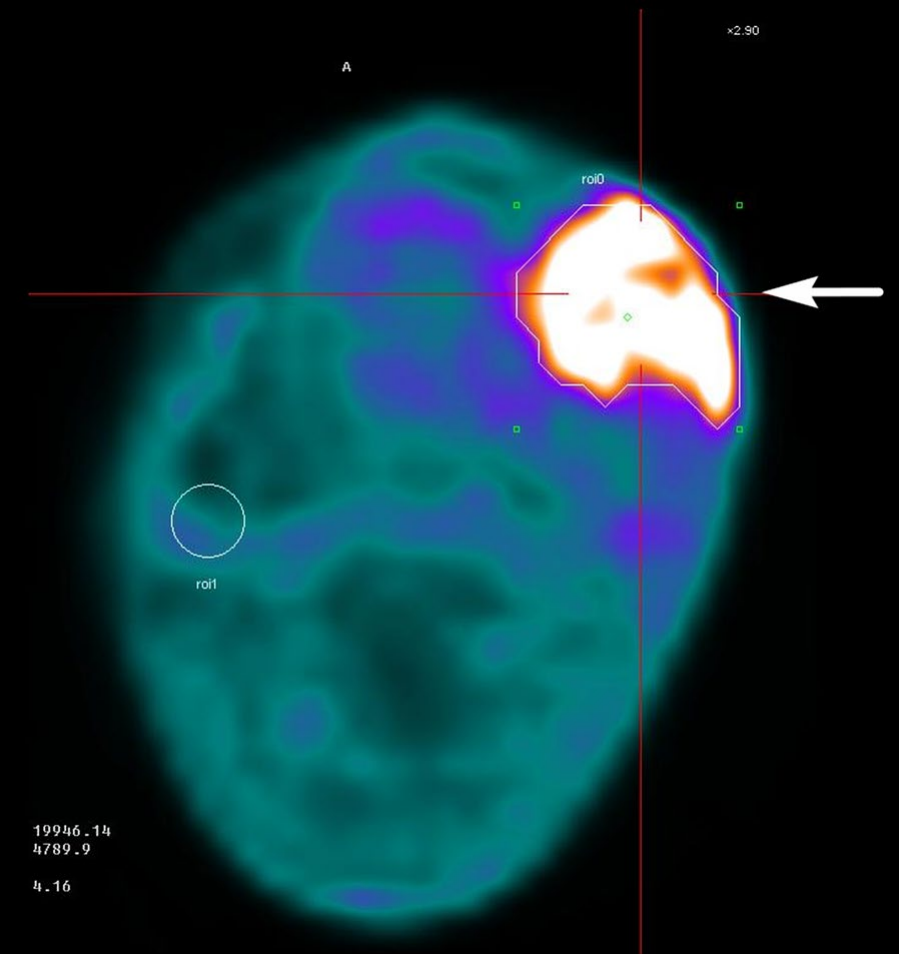
[18]-Fluoro-boronophenylalanine positron emission tomography (^18^F-BPA-PET) of the patient in Fig. 2. The tumor is indicated with an arrow to determine the tumor/normal tissue BPA ratio

In 2008, a clinical protocol with a two-fraction design with adaptive treatment planning was formulated for recurrent HN cancer at the Taipei Veterans General Hospital, a 3000-bed medical facility in Taiwan, China. This was a prospective, non-comparative, open-label, single center phase I/II trial for recurrent HN cancer. The primary endpoints were treatment-related toxicity and tumor response rates. The secondary endpoints were the time to tumor progression, progression-free survival, and overall survival. Inclusion and exclusion criteria have been described in detail in a previous publication [14] and are briefly summarized in Table 1. l-Boronophenylalanine (BPA) was chosen as the boron carrier (Hammercap AB, Stockholm, Sweden and Taiwan Biotech Co. Ltd, Taiwan, China). To increase its solubility, l-BPA was complexed with fructose to form an l-BPA-F solution, which was administered at a concentration of 25 g/L (pH 7.6) [15]. [18]-Fluorine-labeled BPA was used for positron emission tomography (^18^F-BPA-PET) was to calculate the tumor/normal tissue (T/N) BPA concentration ratios (Fig. 3). The treatment planning procedure was previously described in detail elsewhere [16]. Briefly summarized, a CT simulation was performed in the supine position with a slice thickness of 5 mm, and the images were co-registered with T1-weighted MRI and ^18^F-BPA-PET images. The gross tumor volumes (GTVs) and normal structures were delineated and exported to THORplan together with the CT images. Both physical- and biologically-equivalent doses were calculated. The equivalent dose was defined as the sum of the physical dose components multiplied by weighting factors (including relative biological effectiveness and compound biological effectiveness) of each dose component in tissues of the radiation field. The principle prescription dose was approximately 20 Gy-Eq, delivered to 80% of the GTV, in order to limit the dose to the oral mucosa to the lowest possible level. Single portal from anterior, anterior-oblique, or lateral directions were selected according to tumor location. A two-stage injection protocol (180 mg/kg/h for 2 h, and then 90 mg/kg/h for 30 min) was used to maintain a stable blood BPA concentration [17]. Before and during continuous intravenous BPA infusion, blood boron concentrations were measured six times by inductively coupled plasma-atomic emission spectroscopy (ICP-AES) [18] to assess tumor BPA uptake. Prior to the second fraction, tumor contouring was again performed following repeated CT simulation and ^18^F-BPA-PET imaging. The prescription dose was modified accordingly to the second tumor/normal tissue BPA ratio. After BNCT, all patients were followed up regularly with monthly physical examinations, MRI and PET/CT at 3 months intervals following BNCT for 1 year at the Taipei Veterans General Hospital. The follow-up was then continued at longer intervals until treatment failure, death or 5 year survival.

**Table 1:** Inclusion and exclusion criteria for the fractionated BNCT clinical trial

Inclusion criteria	Exclusion criteria
1. Patients with locoregionally recurrent and histologically proved malignancy of the head and neck 2. Prior conventional radiotherapy administered has been given for the disease (except melanoma) 3. Bi-dimensionally measurable disease by MRI and/or CT scan and ≦ 12 cm in the largest dimension 4. Age > 18 and < 80 years, ECOG performance status ≦ 2 5. WBC > 2.5 × 10^9^/L, neutrophil count > 1.0 × 10^9^/L, platelet count > 7.5 × 10^9^/L, serum creatinine < 1.25 × ULN 6. Tumor to normal tissue (T/N) ratio for BPA > 2.5 by ^18^F-BPA PET scan	1. Lymphoma or other tumor type that is expected to respond to chemotherapy or conventional radiation therapy that can be safely given 2. Patients who had an effective standard treatment option like surgery or radiotherapy available 3. Distant metastasis outside the head and neck regions 4. Expected life less than 3 months 5. A time interval less than 3 months from previous radiation therapy 6. Concurrent systemic cancer treatment including chemotherapy or target therapy 7. Pregnancy 8. Restless patients who were unable to lie or sit in a cast for 30–60 min

*BNCT* boron neutron capture therapy, *MRI* magnetic resonance imaging, *CT* computed tomography, *ECOG* eastern cooperative oncology group, *WBC* white blood cell, *ULN* upper limit of normal, *BPA* boronophenylalanine, ^*18*^*F-BPA PET* 18-Fluoro-labeled BPA positron emission tomography

**Fig. 4:**
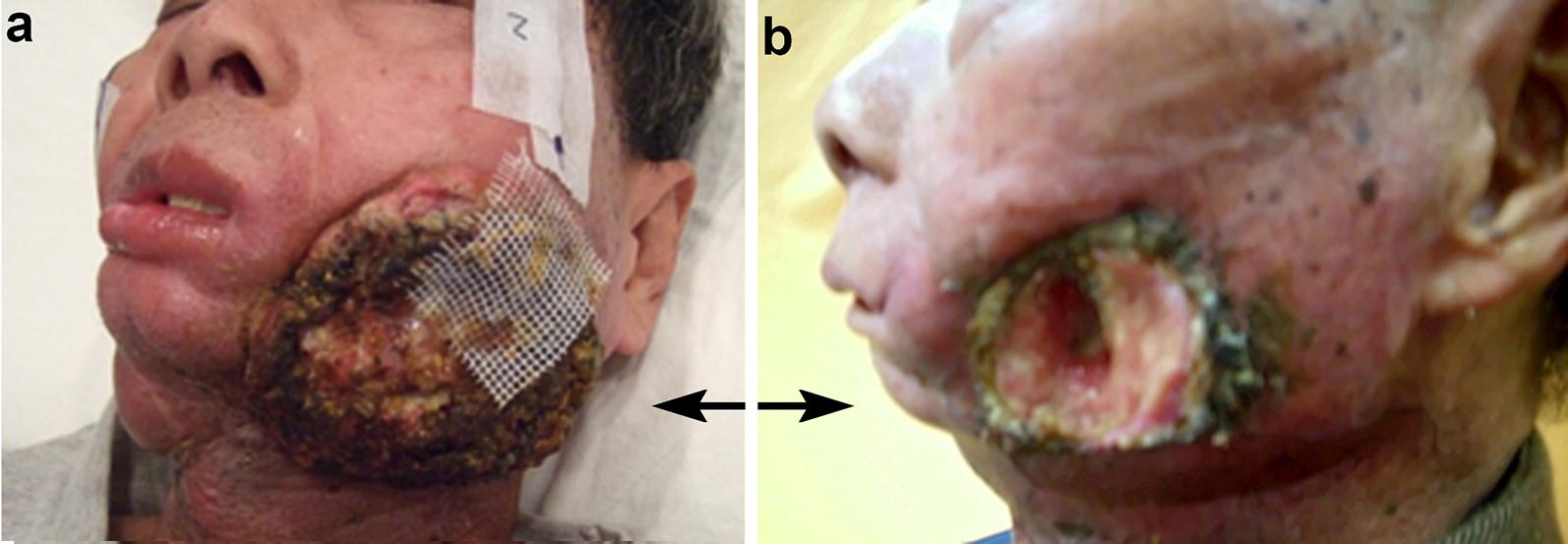
Photos of a recurrent buccal cancer patient before and after boron neutron capture therapy (BNCT). **a** A photo taken before BNCT (recurrent left buccal tumor indicated by arrows). **b** A partial response was found 2 months following two-fraction BNCT. His pain symptoms and quality of life improved significantly

**Fig. 5:**
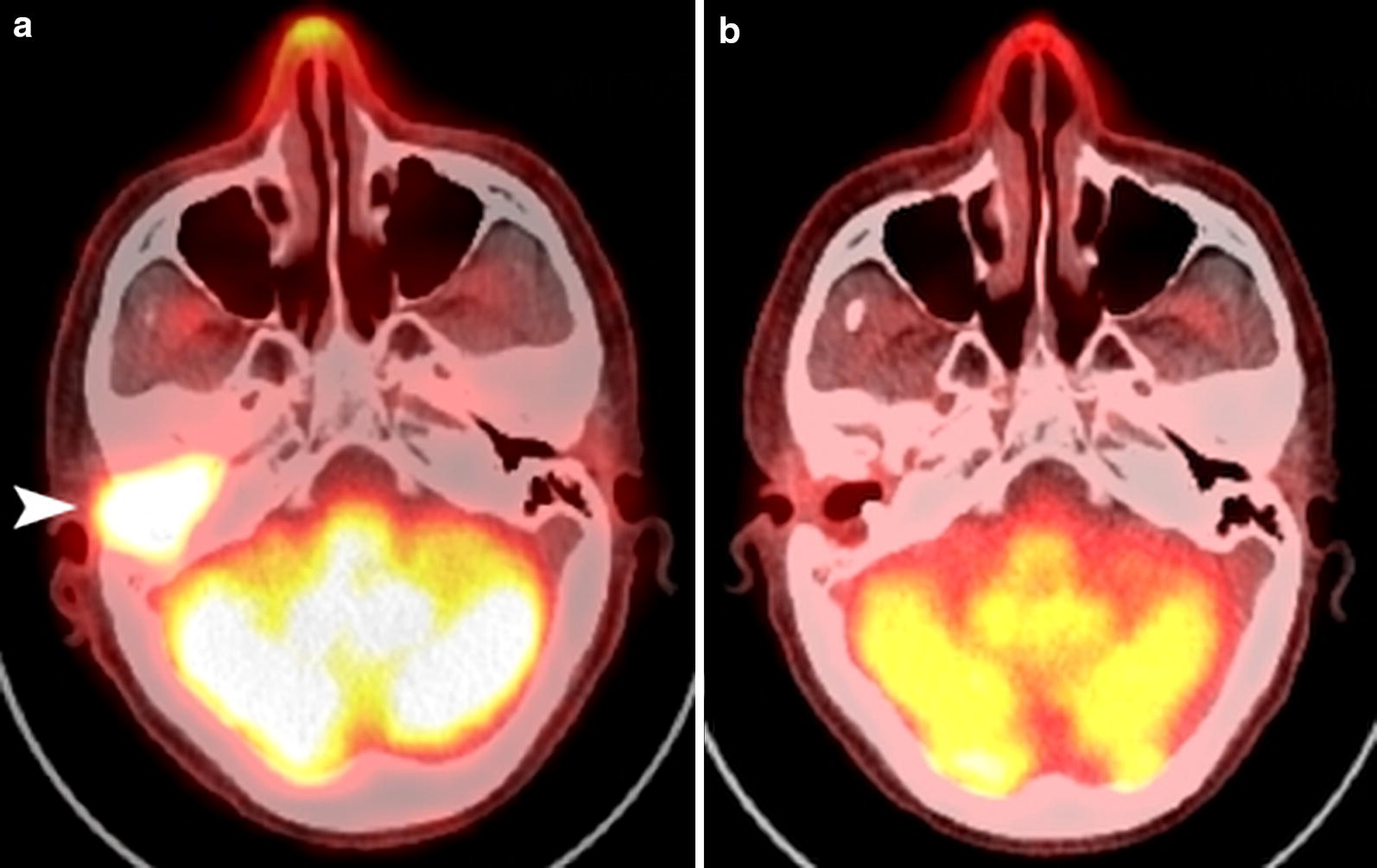
^[18]^F-BPA PET images of a patient with recurrent nasopharyngeal cancer in the auditory canal after two courses of radical radiotherapy and surgery. **a** Before boron neutron capture therapy (BNCT), the bright area anterior to the cerebellum showed the recurrent tumor (arrow head). **b** Five months after two applications of BNCT, the tumor in the auditory canal shrank completely

After the protocol was approved by our Institutional Review Board, 25 recurrent HN cancer patients were screened, among whom, 17 were eligible and subsequently treated with BNCT. All of the patients had been heavily treated with photon radiation therapy of one or two course. The median radiation dose before BNCT was 107 Gy (range 63–165 Gy). The most common primary tumor site was the oral cavity (41%). Demographic data of the patients are summarized in Table 2. The first patient with a recurrent hypopharyngeal cancer, was treated in August 2010. All patients were irradiated in the supine or sitting positions with a support device. During irradiation, polyethylene extension collimators of different diameters covering the GTVs with a safe margin were used to concentrate the neutron beam and facilitate positioning [13, 19]. Fifteen patients received two fractions of BNCT, and two received only one. The median prescription dose of the first fraction was 19.8 and 14.6 Gy-Eq for the second fraction. The median follow-up time was 19.9 months (range 5.2–77.1) months. Nine patients reported improved quality of life after BNCT. The most common acute adverse events were low-grade oral mucositis, radiation dermatitis, and alopecia, all of which were conservatively managed by analgesics and topical steroids. Only one patient with recurrent hypopharyngeal cancer showed grade 4 laryngeal edema and carotid artery hemorrhage. He promptly had a tracheostomy and embolization of the artery. The most common grade 3 late adverse event was cranial neuropathy (two patients). No grade 4 late adverse events were observed. Six patients had complete responses (CRs), as indicated by PET scans at least 3 months after the last BNCT fraction. Photo and image examples of which are shown in Figs. 4 and 5. The other six patients had partial responses, as evidenced by MRI. The 2-year locoregional control rate was 28%, and the 2-year overall survival rate was 47%. Two patients survived longer than 4 years, and both were disease-free at 50 and 77 months after BNCT. The prescription doses, tumor volumes, and responses seemed to be interrelated. Of the 14 patients with tumor volumes < 20 cm^3^, eight (57%) had a CR, and of the six patients receiving a total prescription dose > 40 Gy-Eq, four (67%) achieved a CR.

**Table 2:** Patient demographics and tumor characteristics in the fractionated BNCT clinical trial

Patient no.	Age	Sex	Primary site	Histopathology	Accumulated dose of prior RT (Gy)	Recurrent stage	Tumor diameter (cm)
1	68	M	Hypopharynx	SCC	136.4 in 2 courses	T1N2a	4
2	67	M	Tongue	SCC	66	T2N2a	9.5
3	49	F	Nasal cavity	Sinonasal carcinoma	120 in 2 courses	T4N0	5
4	71	M	Gingiva	SCC	66	T4N0	7.5
5	46	M	Nasopharynx	Undifferentiated ca	136 in 2 courses	T4N0	3.9
6	58	F	Nasopharynx	Non-keratinizing ca	122 in 2 courses	T3N0	2.5
7	57	M	Tongue	SCC	70	T3N0	4.5
8	52	M	Tongue	SCC	70	T4aN0	5.3
9	54	M	Maxillary sinus	SCC	70	T3N0	8.9
10	54	M	Tongue	SCC	63	T0N2b	6.0
11	48	M	Tongue	Spindle cell sarcoma	107 in 2 courses	T4bN0	6.0
12	74	M	Maxillary sinus	SCC	105 in 2 courses	T2N0	3.5
13	51	M	Parotid gland	Adenocarcinoma	136 in 2 courses	T4N0	5.8
14	56	M	Buccal mucosa	SCC	125.9 in 2 courses	T1N0	0.9
15	40	M	Hypopharynx	SCC	95 in 2 courses	T3N0	4.7
16	59	M	Nasopharynx	Non-keratinizing ca	136 in 2 courses	T3N0	3.9
17	62	M	Buccal mucosa	SCC	64	T4aN0	6.4

*BNCT* boron neutron capture therapy, *SCC* squamous cell carcinoma

## Preliminary experience from the second clinical trial (2014–ongoing)

From the experience of our first clinical BNCT trial and other studies [7, 9], re-recurrences near re-irradiated sites were common, despite initially good responses. We hypothesized that by irradiating a larger field around the recurrent GTV with photons, we could obtain better local control IG-IMRT had been used alone as salvage treatment for recurrent HN cancer with acceptable toxicity [4]. Therefore, we developed a new protocol in 2013 combining IG-IMRT with BNCT. The inclusion and exclusion criteria were similar to our previous two-fraction BNCT protocol, except that a longer interval (at least 6 months) was necessary between previous RT and BNCT. BNCT treatment planning was done as prescribed for the first fraction in the previous protocol. For IG-IMRT planning, a selected margin (3–5 mm) around the GTV was added to generate the clinical target volume (CTV), and an additional 3-mm margin was added around the CTV to generate the planning target volume. In two patients, lymphatic drainage areas adjacent to the GTVs were also included in the CTV. Additionally, special attention was paid to limit the dose to critical organs such as the carotid artery, spinal cord, brain stem and mandible. Patients were followed up similar to our previous protocol. After approval of the new protocol in 2014, we recruited 9 eligible patients, 4 of whom had squamous cell carcinomas of the oropharynx, 3 has squamous cell carcinomas of the oral cavity, one mucoepidermoid carcinoma of the parotid gland, and one osteogenic sarcoma of the mandible. The median radiation dose before BNCT was 66 Gy (range 60–102 Gy).

Seven patients were treated with combined therapy, one with BNCT alone due to severe acute toxicity, and another due to old age and the risk of carotid bleeding. The median BNCT doses were, 19.4 Gy-Eq and IG-IMRT: 45 Gy (in 25 fractions). The median follow-up time was 11.7 months (range 4.8–25.9 months). Regarding acute toxicities, almost all had low-grade oral mucositis, radiation dermatitis and alopecia, which were similar to that observed in the previous two-fraction BNCT protocol [14]. Regarding grade 3 toxicity, dysphagia and tumor pain were seen in two patients, infection in one, and facial edema in one. One patient with a recurrent oral cancer showed grade 4 oral bleeding. He was successfully treated by carotid artery embolization. Another patient had grade 4 dyspnea following facial edema and his symptoms subsided following tracheostomy. Three patients showed CR, as evidenced by PET scans 3 months after combined treatment, and another 3 showed PR by MRI. The other 3 had stable disease. The 1-year overall survival rate for all patients was 56%, and one patient was disease-free 25.9 months after combined treatment.

With chemotherapy alone, most patients who are diagnosed with inoperable, locoregionally recurrent HN cancer at previously irradiated sites die of the disease within a few months [3]. Re-irradiation with BNCT with limited toxicity may constitute successful salvage therapy for these patients. In reality, further local recurrence after BNCT is a major cause of treatment failure. There are many possible explanations for local failure following BNCT, including insufficient uptake and non-homogeneous BPA distribution within the tumor, insufficient depth penetration by epithermal neutrons, and an insufficient radiation dose or coverage of the CTV. By combining image-guided fractionated photon therapy with BNCT, we hoped that the second trial would decrease the recurrence rate following BNCT without significantly increasing toxicity. Theoretically, local control should be improved when a larger volume around the recurrent tumor bed (i.e., the CTV) is further treated with photon therapy through IMRT, because the BNCT dose to the adjacent normal tissue would be low. Thus, it may be appropriate to choose an “adequate” margin surrounding the GTV based on previous RT fields, dosages, nearby critical organs, and the risk of re-recurrence. Because the patient numbers of both trials were small and they had disparate primary sites, histopathology, clinical staging, and accumulated radiation doses before BNCT, it was difficult to compare the efficacy and toxicity of the two trials. However, from the dose volume histograms of BNCT and IMRT plans, the dose to normal tissues was often lower with BNCT than with IMRT in the same patient. We hypothesized that better local control with BNCT with combined IMRT, covering a larger tissue volume, at the expense of more severe toxicity, compared to a second application of BNCT. However, it is still too early to draw any conclusions regarding the second trial. Nevertheless, it remains very challenging to treat recurrent HN cancer patients with further radiation, even with a target type of radiotherapy such as BNCT. Recently, promising results from some clinical trials testing systemic treatments, such as molecularly-targeted therapy and immunotherapy for recurrent HN cancer have been reported [20, 21]. In the future, multimodality treatment combining BNCT with new therapeutic approaches such as the use of pulsed ultrasound [22] should be evaluated to avoid excessive normal tissue injury and obtain more durable tumor responses.

## Conclusions

From our experience at THOR, BNCT either alone or combined with fractionated photon radiation resulted in a high response rate for selected recurrent HN cancer patients. Some patients survived more than 4 years, but further locoregional recurrence was the major cause of treatment failure. The few severe complications such as carotid arterial bleeding and laryngeal edema were successfully managed. Finally, we believe that additional modifications of our protocol could lead to even better treatment results for this group of patients.
